# Transactions of the Medical Society of N. C.

**Published:** 1875-10

**Authors:** 


TRANSACTIONS OF THE TWENTY-SECOND ANNUAL MEETING OF THE MEDICAL SOCIETY OF NORTH CAROLINA. Wilson, N. C. : pp. 130.

This volume comes neatly prepared and printed, containing several articles comprehensive and valuable.

